# Temporal changes in species interactions in simple aquatic bacterial communities

**DOI:** 10.1186/1472-6785-12-18

**Published:** 2012-09-17

**Authors:** Minna Pekkonen, Jouni T Laakso

**Affiliations:** 1 Integrative Ecology Unit, Department of Biosciences, P.O. Box 65, FIN-00014 University of Helsinki, Helsinki, Finland

## Abstract

**Background:**

Organisms modify their environment and in doing so change the quantity and possibly the quality of available resources. Due to the two-way relationship between organisms and their resource environment, and the complexity it brings to biological communities, measuring species interactions reliably in any biological system is a challenging task. As the resource environment changes, the intensity and even the sign of interactions may vary in time. We used *Serratia marcescens* and *Novosphingobium capsulatum* bacteria to study how the interaction between resource environment and organisms influence the growth of the bacterial species during circa 200 generations. We used a sterile-filtering method to measure how changes in resource environment are reflected in growth rates of the two species.

**Results:**

Changes in the resource environment caused complex time and species composition-dependent effects on bacterial growth performance. Variation in the quality of the growth medium indicated existence of temporally fluctuating within-species facilitation and inhibition, and between-species asymmetric facilitation.

**Conclusions:**

The interactions between the community members could not be fully predicted based only on the knowledge of the growth performance of each member in isolation. Growth dynamics in sterile-filtered samples of the conditioned growth medium can reveal both biologically meaningful changes in resource availability and temporally changing facilitative resource-mediated interactions between study species. This is the first study we are aware of where the filter-sterilization – growth assay method is applied to study the effect of long-term changes in the environment on species interactions.

## Background

Despite extensive research on resource-consumer dynamics and species interactions between consumers it is still largely unknown how changes in resource environment affect population and community dynamics. Resource environment can change due to abiotic reasons, *e.g.* seasonal fluctuations in weather conditions, but also due to biotic factors including habitat modification by organisms [1,2]. By modifying their environment organisms can change the quantity and possibly the quality of available resources. The two-way relationship between organisms and their resource environment adds complexity to biological systems and makes measuring species interactions a challenging task.

Laboratory microcosms, where bacteria grow on simplified resource environments with only few growth limiting substrates in otherwise controlled conditions, have been useful study systems for resource-consumer dynamics *e.g*. [3-8]. Even initially extremely simple resource environments can rapidly change due to metabolism or other activities of the organisms. Cross-feeding, resource partitioning, and the excretion of secondary metabolites can result in unpredictable changes in the resource composition, which in turn affect the community composition [9,10] or can maintain polymorphic populations [4,10-12]. The increase in environmental or community complexity makes it more difficult to quantify species interactions. Changes in the environmental conditions can change the way species interact. For example, the strength of density-dependent resource competition may change depending on the resource availability [13]. Resource availability may determine whether species experience more intra- or interspecific competition [13]. In this paper we investigate temporally changing, environment-mediated interactions between two bacterial species. We track population size in monocultures and two-species communities over approximately 200 bacterial generations, and measure how temporal changes in the resource environment affect the growth performance of each study species.

Though bacteria are in many ways relatively simple organisms the interaction networks in bacterial community are by no means simple. Complex networks of inhibitory and facilitative interactions often regulate the growth of bacterial strains in communities [14]. Changes in resource quality *e.g*. [15] and evolutionary changes in the interacting species or strains *e.g*. [4,6,9,12,16] can further add complexity to these interactions. The interactions between different bacterial species in aquatic environments are often mediated by the quality of the liquid substrate [17]. Thus, the growth performance of individual species exposed to the liquid substrate reflects the net changes in the environment. In this study we remove bacteria from their growth environment by filter-sterilization, and thereafter measure the short-term growth rate of both study species separately in the filtrate. This methodology is used *e.g.* in applied microbiology in process-related applications [15,18,19], in microbial ecology to test the survival of microbes on different substrates [20], and in evolutionary ecology to test the role of resource modification in evolution of within species diversity [21-23].

In our experiment we use two bacterium species *Serratia marcescens* and *Novosphingobium capsulatum*. These species a) can be cultivated both in isolation and together in laboratory conditions, b) form distinguishable colonies when grown on agar plates, and c) coexist and grow readily on detritus resource used in the experiment [24]. The study species were chosen because they have contrasting growth dynamics [24,25]. *Serratia marcescens* could be loosely categorized as a copiotroph, an organism that can grow rapidly in rich nutritional conditions [26]. Based on growth rates on different resource concentrations *N. capsulatum* is more close to an oligotroph ecotype, which grows well also on low nutrient conditions [26].

The growth medium used in the experiment is filtered plant detritus extract, which consists of several chemically different nutritional resources, potentially allowing niche separation in resource use. Resources are not added to the system during the experiment. Thus, in one species systems our general expectation is that the growth rates of re-introduced species in the filtrate decline towards the end of the experiment. The objective of this study is to test whether the filtering methodology can reveal resource environment mediated species interactions. In a two species system we hypothesize four possible scenarios when species growth rate is measured in medium consumed by the other species (cross-species interaction). First, if species share common resources, growth rate in filtrate is lower than in unconsumed medium. Second, if species do not share resources, growth rate in filtrate is on the same level as in the unconsumed medium. In the third potential scenario growth rates of one or both species are higher in the filtrate than in unconsumed medium. This could be due to asymmetric or symmetric facilitation in resource utilization. Fourth, there could be symmetric or asymmetric inhibition due to resource competition or the secretion of substances that affect bacterial growth. The relaxation of inhibitory effect could also cause higher growth rates in filtrates compared to unconsumed medium.

According to our findings the bacteria *S. marcescens* and *N. capsulatum* exhibit temporally fluctuating interactions, whose strength and direction vary, and which are mediated by the growth medium.

## Results

### Biomass and population size in batch cultures

**Figure 1:**
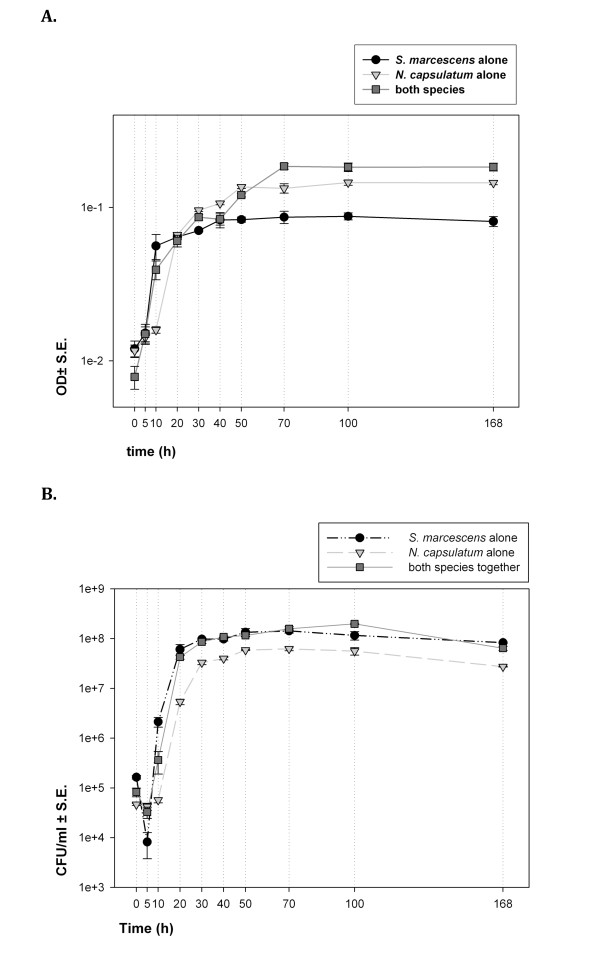
**Biomass and population size during one week. A**) Biomass dynamics as optical density (OD), and **B**) population size as colony forming units (CFUml^-1^) during one week’s growth, when *Serratia marcescens* and *Novosphingobium capsulatum* where grown either separately or together in batch cultures. For the two-species community, the biomass is combined for both species.

**Figure 2:**
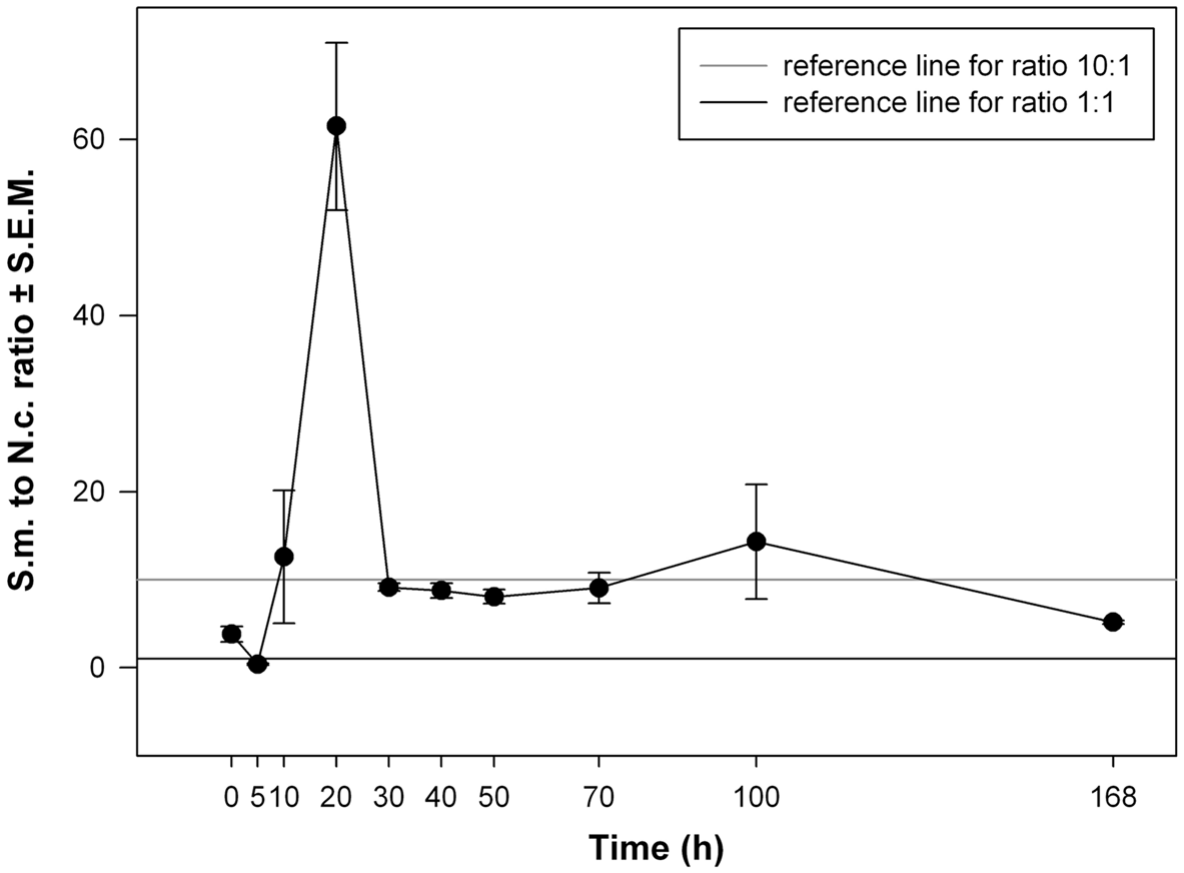
**Species abundances in two-species communities.** Fluctuations in the relative abundance of *S. marcescens* to *N. capsulatum* in two-species communities (based on CFUml^-1^). Reference lines indicate when species abundances are equal (black line at Y-axis = 1), or when *S. marcescens* is ten times more abundant than *N. capsulatum* (grey line at Y-axis = 10).

All populations grew approximately following the logistic growth (Figure 1). Based on the colony forming units (CFUml^-1^) data the time lag before exponential growth phase was circa 5 h for *S. marcescens*, and 10 h for *N. capsulatum* (Figure 1B). After 70 h of growth the total biomass (measured as optical density) reached its peak and was highest in the two-species community (Figure 1A). It was not possible to separate species-specific biomasses in the two-species communities, but when grown separately *N. capsulatum* produced more biomass than *S. marcescens* (Figure 1A). However, based on the CFUs *S. marcescens* had higher population sizes than *N. capsulatum* in isolation (Figure 1B). When *S. marcescens* grew alone the number of CFUml^-1^ declined during the first five hours of growth (Figure 1B). After an initial time lag in growth *S. marcescens* was 10–60 times more abundant in two-species communities than *N. capsulatum* (Figure 2). At the end of the week’s growth the population size of *S. marcescens* was 10^8^ CFUml^-1^ and *N. capsulatum* was 10^7^ CFUml^-1^ both in isolation and in two species communities.

### Growth rates in filtered medium

**Figure 3:**
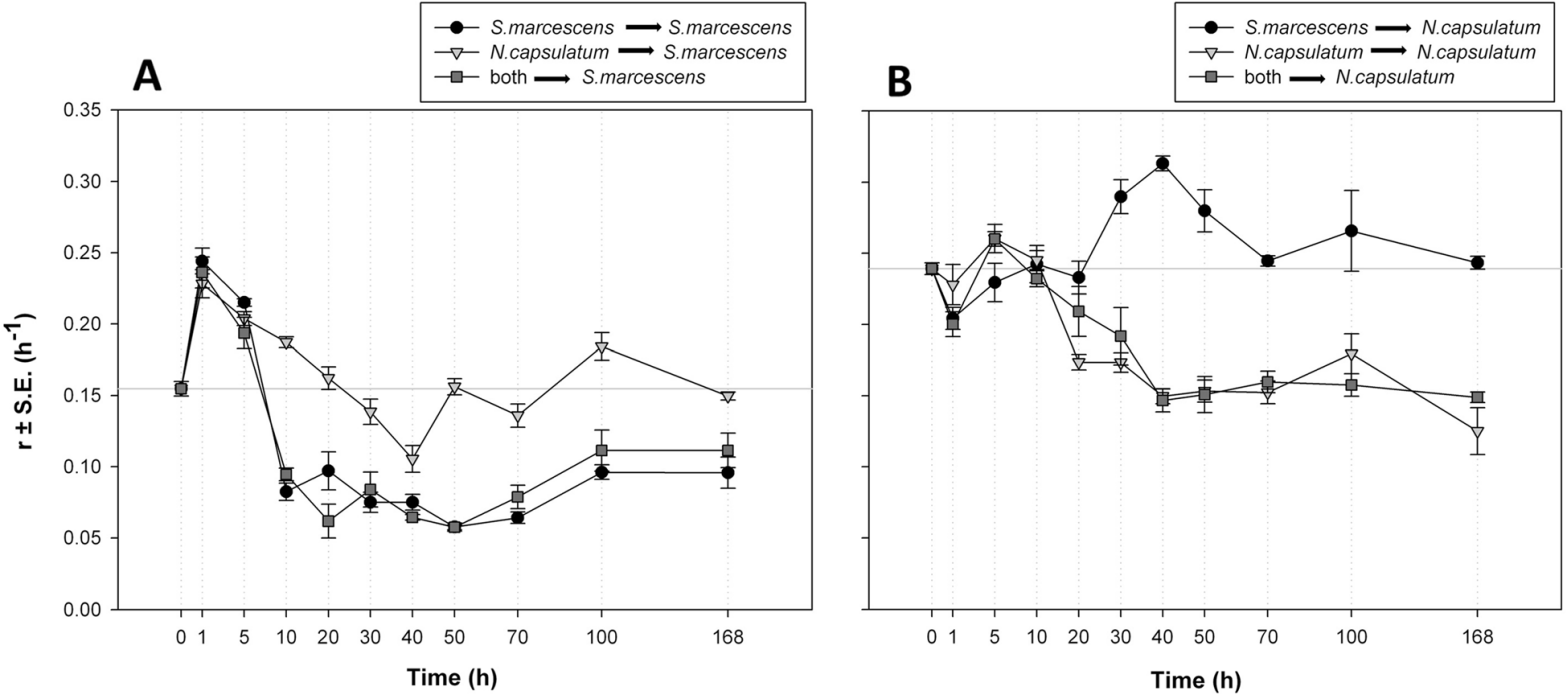
**Growth rates in filtrates.** Maximum instantaneous growth rates (r ± s.e.m.) of **A**) *S. marcescens* and **B**) *N. capsulatum* in filter-sterilized medium. The first species in the legend indicates the consumer species and the second the test species. Consumer species grew in the medium before filtering, and test species’ growth rate was measured in the filtered medium. X-axis is the resource consumption time before filtering, at t = 0 h is the growth rate in unconsumed medium.

*Novosphingobium capsulatum* grew faster than *Serratia marcescens* in unconsumed medium (r ± s.e.m. 0.239 ± 0.004 and 0.155 ± 0.005, respectively; t = -12.89, df = 18, p < 0.001, compare Figure 3A and Figure 3B, at t = 0 h). The growth rates of *S. marcescens* and *N. capsulatum* in consumed medium differed between species and also changed in time (Test sp., Time effect, and their interaction, see Table 1). Moreover, the consumer identity producing the filtrate affected the growth dynamics (Consumer sp., Table 1). Based on the F-values the test species identity had the strongest effect on growth rates (between subjects effects, Table 1).

**Table 1:** Effects of consumer species and test species identity on test species instantaneous maximum growth rates (r)

**Factor**	***df***	***F***	***p***
**Within subject effects**			
**Time**	9	60.5	<0.001
**Time × Test sp.**	18	6.9	<0.001
**Time × Consumer sp.**	9	29.8	<0.001
**Time × Test sp. × Consumer sp.**	18	12.8	<0.001
**Error**	108		
**Between subjects effects**			
**Consumer species**	2	100.0	<0.001
**Test species**	1	1426.6	<0.001
**Consumer sp. × Test sp.**	2	294.3	<0.001
**Error**	12		

“Consumer species” grew in the medium before filtering; “test species” grew in the filtered medium. Results from the factorial repeated measures ANOVA.

**Figure 4:**
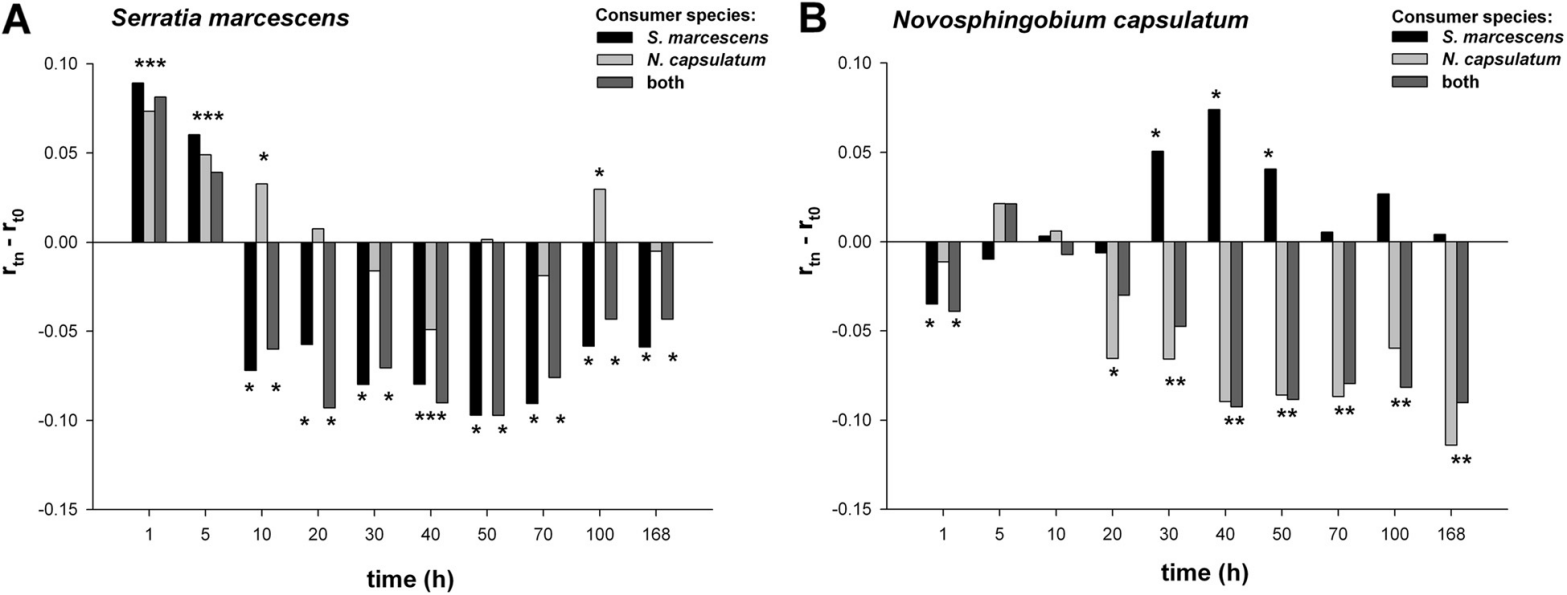
**Growth rates in consumed vs. unconsumed mediums.** Comparison of maximum instantaneous growth rates (r) of **A**) *S. marcescens* and **B**) *N. capsulatum* in un-consumed vs. consumed resource medium. An asterisk indicates statistically significant difference (LSD corrected p < 0.05) between growth rate in unconsumed medium (r_t0_) and in the filtrate at each time (r_tn_), ”time” corresponds to the resource consumption time before filtering, ”consumer species” denotes the species that grew in the medium before filtering.

*Serratia marcescens* grew faster in filtrates from medium consumed for 1–5 h than in unconsumed medium (Figure 4A). Thereafter *S. marcescens* growth slowed down in those treatments where it had grown before filtering. The growth rates of *S. marcescens* were similar in filtrates from monocultures and two-species communities (2-way interaction between time and consumer species F_9,49_ = 1.967, p = 0.064). In cross-species treatments where only *N. capsulatum* had consumed the medium *S. marcescens* grew either faster (sampling times t = 1, 5, 10, and 100 h), slower (t = 40 h), or as well as in unconsumed medium (Figure 4A).

The growth rate of *N. capsulatum* in filtrates from sampling times 5 and 10 h from all treatments was on the same level as in unconsumed medium (Figure 4B). At sampling time 1 h the growth rates were lower than in unconsumed medium if *S. marcescens* had consumed the medium before filtering (Figure 4B). *Novosphingobium capsulatum* grew fastest in filtrates consumed by *S. marcescens* alone for 30–50 h. If *N. capsulatum* had consumed the medium ≥20 h, the growth rates were lower than in unconsumed medium. The presence of *S. marcescens* in two-species communities did not affect the growth rates of *N. capsulatum* in filtrates (2-way interaction between time and consumer species F_9,49_ = 1.981, p = 0.062).

## Discussion

We studied the resource-consumer dynamics in an aquatic bacterial community. In general it is challenging to measure species interactions in natural or semi-natural settings [27], even more so when species have temporally varying effects on the growth environment [1]. The environment of aquatic bacteria typically contains a complex set of food resources and other growth affecting substances and mediates inter- and intraspecific interactions [14,17]. Microbial model systems can give an insight to long-term community dynamics in various environmental settings. To mimic the natural conditions we used as a food resource plant detritus that contains a mixture of substrates. The chemically highly complex plant detritus renders it difficult or impossible to assess how each chemical compound affect growth dynamics or species interactions. However, from the perspective of understanding species interactions the net effect of the resource-mediated interactions on species growth rate can be useful. With a filter sterilization and growth rate assay method we found complex temporal dynamics both in the single-species resource-consumer interaction, and in interactions between consumers in the two-species community. Changes in the growth rates in the absence of resource input after the initiation of the experiment indicate that the species modified the quantity and the quality of the resource environment during the weeklong experiment. Environmental changes that affect growth rates are potentially beneficial to other species in a community, and thus of central importance in determining the community dynamics. We discuss the observed patterns in species interactions and the possible underlying mechanisms.

Yang defines resource pulses as temporal events of increased resource availability with low frequency, short duration, and large magnitude [28]. In that sense batch cultures used in our experiment are pulsed resource-environments with initial resource input with no continuous resource inflow to the study system. In the simplest case the bacterial growth dynamics in a batch culture are expected to follow a resource consumer model with saturating Monod-type growth responses under resource-pulse conditions [29]. In this scenario the population should first follow logistic-like growth and then an exponential decline. In a closed system population decline is inevitable due to resource depletion combined with increased within-species resource competition [17]. Also to the possible accumulation of toxic metabolic by-products can further increase mortality in closed systems [17]. The general pattern of population dynamics of both *S. marcescens* and *N. capsulatum* in the batch cultures included initial rapid population growth rate phase which was followed by a slow decline in population size (Figure 1B). The growing population consumes the resources, which we expected to lead to a monotonically decreasing growth rates in the sterile-filtered samples from the growth media. Indeed, when the consumer (in the batch culture) and the test species (in the filtrate) were the same species the overall growth dynamics did roughly reflect the hypothesised changes in resource availability: after a lag phase the growth rates in filtrates declined below the growth rate in unconsumed medium if the same species had consumed the medium before filtering (Figure 4).

In the cross-species treatments interspecific growth rate measurements revealed temporally changing facilitative interactions in resource utilization and niche differentiation in resource preferences. If species have no overlap in what resource compounds they use from the hay extract medium, the growth rates of the test species should be at the same level in the filtrate as in the unconsumed medium in cross-species treatments. The observation that growth rates of *S. marcescens* in filtrates, where *N. capsulatum* had been the only consumer species for 10 h or more, were on the same level as in unconsumed medium could be due to differences in substrate preferences. We found also indications of facilitative interactions where species benefit from the metabolic activity of the other: *N. capsulatum* grew better in the medium consumed by *S. marcescens* for 30 to 50 h than in unconsumed medium. Facilitative interactions in resource use are potentially common in microbial communities, and the interaction between community members makes the utilization of a complex resource more efficient [30]. The hay extract medium contains recalcitrant resource particles like lignin and cellulose that are more difficult for bacteria to utilize than for example glucose. Extracellular enzymes can expedite resource uptake by breaking up recalcitrant substrates. *Serratia marcescens* is capable of secreting exoenzymes that either break down the resource particles, or increase the rate of nutrient uptake [31]. The increased growth rates in the conditioned medium could indicate between-species facilitation resulting from accumulation of extracellular enzymes [14,17].

Enzymatic activity of bacterial cells can also affect the length of the lag phase. Lag phase is typical for microbial growth dynamics. The length of the lag phase is dependent on the physiological stage of the cells and environmental conditions, and varies between species. Some minimum level of enzymatic activity may be necessary before the onset of fast growth phase. In general, during the lag-phase cells adapt physiologically to changes in environmental conditions, and cell size increases prior the cell division [17]. In the batch cultures both species had a lag phase before the onset of fast population growth. The rate of enzyme production can also partly determine how long the lag phase is. *Novosphingobium capsulatum* had longer lag phase than *S. marcescens*, possibly reflecting differences in enzymatic activity between these species. Longer lag phase could be also due to slower physiological adaptation to culture conditions. Furthermore, the growth rates of *S. marcescens* were higher in all filtrates from the first 5 h than in unconsumed medium regardless of which species grew in the medium before filtering. Thus, *S. marcescens* can utilize not only resources that are modified by it’s own enzymatic activity but also substrates that are modified by of *N. capsulatum*. The type strain of *N. capsulatum* is able to assimilate *e.g.* D-cellobiose, fumarate, and gluconate (detailed list in Tiirola *et al*. [32]), and based on our observations can grow better on low resource concentrations than *S. marcescens*[25]. It seems that both species do modify their resource environment at the beginning, and these changes are immediately beneficial to *S. marcescens*, but not to *N. capsulatum* which is consistent with the observed species-specific time lags before exponential growth phase in the population growth in the batch cultures. Furthermore, the enzymatic activity that enables rapid population growth could be defined as a habitat modification process [2,33]. In this system it may not be species specific, but instead resource partitioning and enzymatic activity of one bacteria species are potentially beneficial to other species also. A more detailed analysis of changes in chemical composition of the filtrates is needed to disentangle the exact mechanisms in resource utilization we observed between *S. marcesens* and *N. capsulatum*.

We postulated that within one week’s time the resource levels in a batch culture inevitably would go down, and bacteria experienced both “feast and famine” periods [24]. In the batch cultures both *S. marcescens* and *N. capsulatum* were able to maintain high population size without notable population crash until the end of the one-week long experiment. Based on the short-term growth rates in filtered samples, the resource level reaches treatment specific plateau after the fast growth phase. However, during the experiment the resource levels did diminish, which is seen in the slower growth rates in filtrates where consumer and test species were the same. Bacteria have several mechanisms that enable their survival in low resource conditions after a pulse-like resource inflow [22,34]. When resources are scarce some bacteria can turn to scavenging or cannibalism [22]. In addition, during the one-week long experiment evolutionary changes which enable metabolizing new substrates from the medium and / or cross-feeding are likely to occur [9,12,14,16,35]. Furthermore, niche differentiation in any resource potentially leads to evolutionary changes in bacterial strains [36]. In this experiment the focus was not in evolutionary changes in bacterial strains, but temporal changes in the resource availability. The test species in the short-term growth rate measurements had to be comparable between treatments and measurement times, and for that reason we chose to use stock strains, *i.e.* ancestral bacterial strains, not the strains contemporaneous with the filtrate from resource medium. Thus, the observed changes in growth performance in filtrate are due to qualitative changes in the growth medium, not due to potential evolutionary changes in bacterial strains [24].

In general, our results suggest that *S. marcescens* modifies the resources environment, which enables the rapid increase in population size after the initial lag phase. The effect of resource modification can be seen already after one hour in the high instantaneous growth rates in filtrates. *Novosphingobium capsulatum* potentially also benefits from some unspecified metabolic by-products of *S. marcescens*. The interspecific interactions found in our study highlight the idea that one bacterium’s trash is another one’s treasure. This ability to utilize the metabolic waste products of other species potentially promotes diversity in microbial communities [9,12,22] and can enable long-term coexistence [24]. When grown together in a pulsed resource environment these species coexisted throughout a 13 weeks long experiment [24]. The comparison of growth dynamics in filtrates from one- and two-species treatments reveals that these two species might not share the same resources in the complex medium. If these species competed for resources, this type of beneficial effect of *S. marcescens* on *N. capsulatum* might support the long-term coexistence of the species. However, when species grow together, *N. capsulatum* does not have any obvious benefit such as increase in biomass or population size from the presence of *S. marcescens*. Actually, *S. marcescens* was at minimum ten times more abundant in the two-species community throughout the week-long experiment. If these species share common resources, the intraspecific resource competition is likely to be stronger than interspecific in our study system.

Two aspects that add niche heterogeneity in batch cultures, and therefore can affect the species abundance ratio and growth dynamics, are oxygen availability and biofilm formation. Difference in sensitivity to anaerobic conditions could affect the species growth ability. Both study species grow in aerobic conditions, but only *S. marcescens* can grow also in anaerobic conditions [37,38]. Provided that anaerobic conditions occur, it would benefit *S. marcescens* in the two-species communities. Anaerobic conditions are, however, highly unlikely in our experiment setting, as the plant detritus is not in all parts rapidly decomposable and the concentration was extremely low (2.15 mgl^-1^) and the surface to volume ration in our systems quite large facilitating aeration even in static cultures. Both study species are able to form biofilm (*S. marcescens*[39], *N. capsulatum* (Pekkonen, unpubl. data)). Especially towards the end of the experiment there was apparent biofilm formation within the batch cultures. In practice, it is close to impossible to avoid biofilm formation in bacterial microcosms experiments. Bacteria form biofilm not only in static batch cultures such as we used but also in batch cultures on orbital shaker [40], and in flow-through chemostats [41]. The biofilm formation increases niche heterogeneity, which in turn can promote niche differentiation, and affect the competitive interaction within and between species [41]. It is not known whether these two species would differentially benefit from biofilm formation, or whether this hypothesised difference would affect the conclusions on the filtrate growth rate assay with suspended bacteria we used. However, in the initial phases of the experiment the biofilm could not have played any confounding part as the experiment was started with suspended cells.

Our growth performance assay in filter-sterilized consumed growth medium is a proxy of the overall, biologically meaningful effect of temporal changes in the environmental conditions. Similar methodology has been used in studies of cannibalism or cross-feeding interaction between different strains of *E. coli*[22,23], and in studies assessing the significance of different bacterial species in a cellulose degrading process [19]. This is the first study we are aware of where the filter-sterilization – growth assay method is applied to study the effect of long-term changes in the environment on species interactions.

## Conclusions

The growth rate measurements in the medium filtrates from different time points demonstrate temporally changing, species-dependent within-species inhibition and facilitation dynamics. The simplest resource-consumer models cannot predict these dynamics. The temporal changes in growth rate, and the relatively high growth rates of test species in filtrates from media, where resource concentration should already be low, both highlight the significance of understanding the dynamics of the non-living resource environment and the habitat modification of organisms.

## Methods

### Summary of the experimental setting

We conducted a factorial batch culture experiment to test how resource consumption time and identity of the consumer species affect growth rates of bacteria feeding upon filter-sterilized growth medium. Buffered cereal leaf extract was inoculated with either *Serratia marcescens*, or *Novosphingobium capsulatum*, or both species. Population size in batch cultures was measured both as the number of living cells and as total population biomass. Filter-sterilized samples were taken from the growth medium at different times. In separate short-term measurements the growth rate of both species in all filtrated media was measured based on changes in optical density.

### Study organisms

*Serratia marcescens* (from American Type Culture Collection strain ATCC 13880) and *Novosphingobium capsulatum* (ATCC 14666) are gram-negative, rod shaped bacteria that do not form spores. *Serratia marcescens* is facultatively anaerobic, typically 0.3–1.0 × 1.0–6.0 μm bacterium, and belongs to the family of Enterobacteriaceae [37,38]. The ATCC strain of *S. marcescens* was originally isolated from pond water. *Novosphingobium capsulatum* is aerobic, 0.3–0.5 × 1.0–3.0 μm size bacterium, and belongs to the family of Sphingomonadaceae [42]. The *N. capsulatum* strain was originally isolated from distilled water [42,43]. Species can be easily separated based on colony morphology: *S. marcescens* forms white, pink or red colonies, *N. capsulatum* forms yellow colonies when grown on Nutrient Broth agar plates. Both species can be found in aquatic environments, and they also grow readily on cereal leaf medium used in our experiments. The species have different growth responses to fresh cereal leaf medium: *N*. *capsulatum* grows faster on low concentration than *S*. *marcescens*. *Serratia marcescens* however grows faster on intermediate and high concentrations (mean ± s.e.m Monod parameters estimated from measured growth rates in 0.1–1.0 gl^-1^ hay extract are: maximum growth rate r_max_ = 0.103 ±0.047, half saturation constant K_s_ = 0.29 ±0.36, and r_max_ = 0.418 ±0.157, K_s_ = 1.72 ±0.89 for *N. capsulatum* and *S. marcescens*, respectively [25]).

### Batch culture preparation and sampling

Bacteria were grown as batch cultures in buffered medium containing 1 gl^-1^ of cereal leaf powder (Ward’s natural science, Rochester, NY). The microcosms were filter-capped 250 ml cell culture bottles (Corning) containing 150 ml of the medium. The medium was prepared as follows: 1 gl^-1^ of cereal leaf powder in deionised H_2_O was boiled for 10 min, cooled down and filtered through a glass microfibre filter (GF/C, Whatman). The filtering procedure leaves 2.15 mgl^-1^ dry weight of cereal leaf powder to the final medium. Phosphate buffer adjusted to pH 7.5 [1.57 g of K_2_HPO_4_·3 H_2_O, 0.4 g of KH_2_PO_4_, 0.5 g of (NH_4_)_2_SO_4_, 0.1 g of MgSO_4_·7 H_2_O, 0.01 g of NaCl, and 0.023 g of CaCl_2_·2 H_2_O per 1 l of deionised H_2_O] was added to the medium. The medium was autoclaved at 121°C for 20 min. The medium was shaken before autoclaving and before separating the medium into the culture bottles.

Bacteria were cultivated for three days on agar plates (10 g of nutrient broth (Difco™, BD), 2.5 g of yeast extract (Scharlau Chemie S.A.), and 15 g of agar (Scharlau Chemie S.A.) in 1 l of deionised H_2_0) prior to inoculation to the growth medium. Approximately 50 colonies were streaked from an agar plate, and mixed in sterile phosphate buffered deionised H_2_O. All microcosms received equal total biomass of the inoculum (210 μl, optical density 0.06, when measured with wavelength 595 nm, which equals 5.6 × 10^6^ ±1.3 × 10^6,^ and 3.4 × 10^6^ ±4.4 × 10^5^ CFUml^-1^ ± s.e.m. of *S. marcescens,* and of *N. capsulatum*, respectively). For the two-species community the species were mixed in 1:1 ratio. All treatments had three replicates. The microcosms were kept at 25°C. The relatively low concentration of the detritus resource and the volume to surface area ratio of the microcosms (volume 150 ml, surface area c. 42 cm^2^) suggest that oxygen was available throughout the experiment in all parts of the microcosms.

Growth medium was sampled 1, 5, 10, 20, 30, 40, 50, 70, 168 h after inoculation. At each time three samples were taken: living cells for population size estimation, growth medium for biomass measurements, and filter-sterilized growth medium for the separate short-term growth rate measurements. Due to practical reasons biomass and population size measurements were done later from frozen samples, not immediately after the sampling. We tested that the cell growth was similar before and after the freezing procedure.

### Population size in batch cultures

At each sampling 0.5 ml of the medium was aseptically transferred and mixed to 0.5 ml of sterile freezing solution and stored in -70°C. Freezing solution contains nutrient medium [10 g of nutrient broth (Difco™, BD), 1.25 g of yeast extract (Scharlau Chemie S.A.) in 1 l of deionised H_2_0] and glycerol (bidistilled 99.5% W/V, WVR) in 1:5 volume ratio. Population sizes were estimated from thawed samples based on standard serial dilution plating procedure. Sterile buffered deionised H_2_O was used to dilute samples. Before counting the colony forming units, bacteria were grown for three days on agar plates [1 g of nutrient broth (Difco™, BD), 2.5 g of yeast extract (Scharlau Chemie S.A.), and 15 g of agar (Scharlau Chemie S.A.) in 1 l of deionised H_2_0] in 25°C temperature. Number of colony forming units per millilitre (CFUml^-1^) of original growth medium was calculated per plating. The overall estimate of CFUml^-1^ in each experimental unit per sampling time was counted as a weighted mean of all platings per sample where the plating dilution coefficient was used as the weight. In serial dilution plating the precision of the CFUml^-1^ estimate is lower, the higher the dilution coefficient. We used weighted mean instead of arithmetic mean to reduce the overestimation error, which could result from plating where number of colony forming units is low and dilution coefficient high [44]. Data on CFUml^-1^ are also used in a separate long-term experiment [24].

### Biomass in batch cultures

0.5 ml of growth medium was stored in -20°C for biomass measurements. Biomass at each sampling time was measured as optical density (OD) at 420–580 nm with Bioscreen C spectrophotometer (Growth Curves AB Ltd, Vantaa, Finland). Biomass was calculated as the total OD minus background OD of the filtered growth medium per each replicate population, treatment and sampling time.

### Growth rates in the filtered medium

At each sampling 3–6 ml of the growth medium was first filter-sterilized (0.22 μm, Millex, MCE 33 mm 50S), and then stored in -20°C to be used later in the growth rate measurements. Filtered samples from the growth medium were used as substrate to measure bacterial growth in medium consumed by the same species, the other study species or both two species. The growth rates in the filtrate were measured as OD at 420–580 nm with Bioscreen C spectrophotometer at 5 min intervals. The filtered growth medium sample was thawed and 360 μl of the filtrate and 40 μl of either *S. marcescens* or *N. capsulatum* inoculum (OD = 0.25) was inserted into each well of the microtiter plate (Honeycomb 2, Thermo Electron Oy). 4–7 subreplicates per sample were used in the growth rate measurements. The inoculum containing growing bacteria was prepared as in the batch culture experiment. The relatively large inoculation volume was used to ensure that the population size was detectable immediately after inoculation, and the maximum growth rate measurement could start as early as possible. The maximum instantaneous growth rates were found on average 32 ± 0.75 min ± s.e.m. after the measurement started. The use of short duration of OD measurements (<2.5 h), and inoculation with actively growing bacterial mass was aimed to reduce the possibility that changes in the growth medium occur during the measurement, or that accumulation of dead cell biomass significantly affected the growth rate estimation.

### Effect of filtration

There is a possibility that the filtration and freezing could mechanically break down resource particles larger than the filter pores. This could result in a change in the composition of the medium compared to the unfiltered medium, and possibly affect the resource availability. To quantify this effect, we measured the growth rate of each studied species in unconsumed hay extract medium which was either filtered or filtered and frozen in similar fashion as the samples from the experiment, and in control medium without the filtering and freezing procedure, 10 replicates per each. The growth rate measurements in unconsumed medium were done similarly as the measurements in filtrates from the consumed medium. When the unconsumed medium was filtered and frozen at -20°C, and then thawed, the growth rates were higher than in untreated medium. The mean difference in the level of growth was 0.020 ± 0.006 (mean ± s.e.m) for *S. marcescens* (t = -3.213, df = 18, p = 0.005), and 0.037 ± 0.006 for *N. capsulatum* (t = -6.053, df = 18, p < 0.001). Separate experiments (results not shown) suggest that apart from the elevation of growth, there were no significant time dependent interactions with the filtering effect on the studied species. As we have no indication that there were filtering-induced systematic interactions between measurement times and species identities, we use the filtering method derived r values to interpret the species interactions.

### Data analysis

Growth rates in the filtrates were calculated as the slope of the linear regression of natural logarithms of the background-corrected optical densities versus time. The maximum instantaneous growth rate was taken as the point where the slope of the linear regression reached its highest value. The length of the time-window used in the fitting procedure was 100 min (20 data points). The replicate measurements (n = 4) of growth rate in the filtrate were averaged for each microcosm. Data were analysed as factorial repeated measures ANOVA. The model included growth rate as a response variable, sampling time as a repeated within subject factor, and consumer species and test species identity and their two-way interaction as between subject factors. The measurements in unconsumed medium (t = 0 h in Figure 3) were not included in the repeated measures ANOVA.

The pair-wise comparisons between growth rate in filtrates from different sampling points and treatments, and growth rate in unconsumed medium were done using univariate ANOVA for each test species and treatment combination separately, growth rate as a response variable and time as a factor. LSD post-hoc test was used for pairwise comparisons (see Figure 4). In these comparisons we used as the t = 0 h growth rates measurements done in filtered and frozen unconsumed medium. The effect of sample handling on unconsumed medium (the effect filtering and freezing), and comparison of growth rates between test species in unconsumed medium were analysed using 2-tailed t-test. Whether the monoculture vs. two-species community treatment had effect on growth rates was analysed for both test species separately using univariate ANOVA. Model included growth rate as a response variable and consumer species, sampling time, and their interaction as fixed factors. Data were checked to be suitable for the assumptions of the above tests and the statistical analyses were done using SPSS v.16.0.1. (SPSS Inc., Chicago, IL).

## Abbreviations

CFU: Colony forming units; OD: Optical density.

## Competing interests

Neither of the authors has competing interests.

## Authors’ contributions

MP and JL designed and co-ordinated the study. MP conducted the experiments and collected and organised the data. JL and MP designed and MP performed the statistical analyses. MP drafted the manuscript. JL supervised the research and revised the manuscript. Both authors read and approved the final manuscript.
